# A comparative analysis of body composition assessment by BIA and DXA in children with type II and III spinal muscular atrophy

**DOI:** 10.3389/fneur.2022.1034894

**Published:** 2022-11-18

**Authors:** Wenqiao Wang, Yijie Feng, Qi Long, Fei Chen, Yuzhi Chen, Ming Ma, Shanshan Mao

**Affiliations:** ^1^Department of Clinical Nutrition, The Children's Hospital, Zhejiang University School of Medicine, National Clinical Research Center for Child Health, Hangzhou, China; ^2^Department of Neurology, The Children's Hospital, Zhejiang University School of Medicine, National Clinical Research Center for Child Health, Hangzhou, China

**Keywords:** spinal muscular atrophy, body composition, dual-energy X-ray absorptiometry, bioelectrical impedance analysis, comparative analysis

## Abstract

**Background:**

Body composition analysis is a valuable tool for assessing and monitoring the nutritional status of children with spinal muscular atrophy (SMA). This study was designed to compare the consistency of bioelectrical impedance analysis (BIA) and dual-energy X-ray absorptiometry (DXA), as the gold standard method for assessing body composition in clinical practice when treating children with type II and III SMA.

**Methods:**

From 2019 to 2021, we performed a retrospective analysis of body composition by DXA and BIA measurement methods in patients with type II and III SMA treated at a Chinese tertiary children's hospital. Fat mass (FM), muscle mass (MM), bone mineral content (BMC), and visceral fat area (VFA) were compared using paired sample t-tests. We calculated Lin's concordance correlation coefficient (CCC) and Spearman correlation coefficient to verify the correlation between DXA and BIA measurements. Bland–Altman analysis was used to assess the consistency of the two methods.

**Results:**

Fifty-seven children with type II and III SMA were recruited. Compared with body composition measured by DXA, the average FM measured by BIA is significantly lower (*P* <0.001), whereas the average MM, BMC, and VFA measured by BIA are significantly higher (*P* < 0.001) in children with SMA. Overall, the difference between MM (Delta [BIA-DAX] = 1.6 kg) and FM (Delta [BIA-DAX] = −1.6 kg) measured by DXA and BIA was minor, whereas the difference of VFA (Delta [BIA-DAX] = −43.5 cm) was significantly large. Correlation analysis indicated a substantial correlation of MM (CCC = 0.96 [95% confidence interval (CI) = 0.93–0.98], *r* = 0.967 [*P* < 0.0001]) and FM (CCC = 0.95 [95% CI = 0.92–0.97], *r* = 0.953 [*P* < 0.0001]), and poor correlation of BMC (CCC = 0.61 [95% CI = 0.42–0.75], *r* = 0.612 [*P* < 0.0001]) and VFA (CCC = 0.54 [95% CI = 0.33–0.70], *r* = 0.689 [*P* < 0.0001]) measurements between the two methods. The Bland–Altman analysis suggests that the majority of participants were within LOA. In addition, differences in MM and VFA measurements between BIA and DAX increased according to patients' increasing height, whereas differences in FM and BMC did not differ with height.

**Conclusion:**

BIA overestimates MM and underestimates the FM, BMC, and VFA in children with SMA compared with DXA measurements. Overall, the non-invasive, easy-to-use, and repeatable BIA measurements were found to be in good agreement with DXA measurements, especially for FM and MM, which are essential parameters for the nutritional evaluation of children with SMA.

## Introduction

Spinal muscular atrophy (SMA) is an autosomal recessive neurodegenerative disease mainly caused by the homozygous mutation or deletion of chromosome 5q in the survival motor neuron 1 gene. This mutation causes a deficiency in survival motor neuron proteins (1). Clinically, patients with SMA present with progressive muscle atrophy and weakness, which can result in difficulties in swallowing and feeding and respiratory failure (2). SMA is generally considered as a primary lethal genetic disease in infants, affecting one in every 6,000–10,000 newborns globally (3, 4). Based on the age of disease onset and maximal motor function, SMA can be classified into four subtypes, ranging from type I (the most severe form) to type IV (the mildest form) (5, 6). Type I SMA is defined as infants with disease onset before the age of 6 months who cannot sit independently. Type II SMA refers to children with disease onset between the ages of 6–18 months who can sit independently but cannot walk independently. Individuals with type III SMA can walk at some point in their lives, even if they lose independent ambulation later. Individuals with type IV SMA can walk unassisted after onset in late childhood or adulthood. The most common disease types in surviving children are types II and III (7).

The current updated consensus statement for the standard of care in SMA children highlighted multidisciplinary team (MDT) care is devoted to reducing complications and potentially improving patients' and caregivers' quality of life (5, 8, 9). Furthermore, it emphasizes the importance of individualized nutrition support, which is probably indispensable given the rapid development of gene-targeted and disease-modifying drug therapies (10, 11). Nevertheless, assessing body composition using weight or body mass index (BMI) may be misleading because children with SMA have imbalanced muscle mass (MM) and fat mass (FM), which contribute to inaccurate individually designed energy prescriptions based on the results measured using estimating equations (12, 13). Accurate assessments of body composition are critical components of comprehensive nutritional assessments and have been proven to guide individualized nutrition management and help improve clinical outcomes (5, 12, 14).

There are numerous methods for determining body composition, ranging from simple indirect measures, such as calipers (skinfold thickness), to sophisticated and noninvasive instruments, such as computed tomography (CT), ultrasound, bioelectrical impedance analysis (BIA), and dual-energy X-ray absorptiometry (DXA) (15, 16). Hoffer Nyboer and Thomasset pioneered BIA in the 1950s and 1960s (17, 18), and since then, BIA has been widely used to measure body composition in medical institutions (19). BIA measurements rely on the principle that the body's water, FM, and MM have different impedance or resistance values for a small electric current, with lower impedance in adipose tissue and higher impedance in the muscle area (16). Several BIA devices have been developed to quantify the number of electrical frequencies [e.g., single frequency (SF-BIA) and multifrequency (MF-BIA)]. MF-BIA has higher accuracy and reliability for estimating body composition and can estimate bone mineral content (BMC) (20).

Despite the limitations and confounding influences of all body composition methods, DXA is frequently regarded as a reference method for evaluating BMC, FM, and MM (21, 22). However, DXA has limitations because it is not portable and expensive and frequently requires training and operation by licensed technicians because of the small amount of potential radiation exposure. In contrast, BIA is relatively simple to operate, quick, inexpensive, non-invasive, and can be used in most environments without the need for highly trained personnel.

Previous studies have shown that BIA has a good consistency for measurements of fat mass in both healthy and obese people (23, 24). Differences between BIA and DXA measurements in patients with neuromuscular disease have rarely been explored. Therefore, this study was designed to investigate the correlation of body composition measurements between DXA and MF-BIA devices in children with type II and III SMA.

## Materials and methods

### Participants

We retrospectively collected data for children with type II and III SMA from the Children's Hospital of Zhejiang University School of Medicine between December 2019 and September 2021, following the inclusion criteria listed as follows: (1) Confirmed diagnosis of 5q SMA with type II or III by genetic testing, (2) Ages ranged from 3 years to 18 years, (3) Both DXA and BIA examinations were completed.

All the patients who participated in our study did not take any disease-modifying treatment or take part in any experimental pharmacological trials at the time of recording. Patients who were diagnosed with type I SMA or with incorrect and inaccurate data were excluded before analysis.

Medical details such as demographics, puberty status, disease characteristics, ambulation status, and body composition with DXA and BIA were collected using a full medical record chart review from the patient's visit to the outpatient SMA MDT clinic. Patients' motor function was evaluated based on Hammersmith Functional Motor Scale–Expanded (HFMSE) score (ranged from 0 to 66). This study was approved by the Ethics Committee of the Children's Hospital of Zhejiang University School of Medicine (No. 2019-IRB-171). Informed consent was obtained from the patients or their guardians before their enrollment in this study.

### Anthropometric measurements

Body weight (BW), height, BMI, and body composition with BIA were measured as part of a nutritional assessment by trained dietitians during a standard evaluation. The height was replaced with arm span in non-ambulatory patients using a flexible non-stretchable tape. BW was specifically measured with the assistance of caregivers. The children and their caregivers were weighed together, and then separately, and the difference in the two measurements was used to calculate the subject's weight. BMI (kg/m^2^) was determined. The World Health Organization Anthro Plus software was used to calculate the height for age Z score (HAZ) and BMI Z score (BAZ).

### Body composition

Each subject's body composition was measured after a 12-h fast, using a DXA (Model: Hologic Horizon W, Hologic Inc, Danbury, CT, USA) and a Inbody 770 MF-BIA device (InBody S10, Cerritos, CA, USA) under the standardized conditions required in the morning. Quality control calibration procedures for DXA were performed on a spine phantom. The weight of each subject was determined using a calibrated scale. The children were instructed to maintain a supine position on DXA within the scanning table's borders, and each body scan took approximately 10 min. The subjects remained standing barefoot on the specific inspection platform while their middle fingers, thumbs, and ankles were clamped with corresponding detector electrodes for the InBody S10 BIA. For patients unable to stand independently, they needed to sit in a nonmetallic chair to receive measurements. MM, FM, BMC, and VFA measurements were obtained using both DXA and BIA. All procedures were performed within 1 h on the same day of the visit, during which the children did not consume any foods or fluids.

### Statistical analysis

The mean difference between BIA and DXA measurements was compared using the paired sample *t*-test. The mean difference of 1.96 SD was taken as the indicator to calculate the 95% limits of agreement (LOA) for the difference in measurements of the same individual (25). We calculated Lin's concordance correlation coefficient (CCC) and a 95% confidence interval (CI) to evaluate the agreement of the two methods (BIA and DXA) in patients with SMA and subgroups with two SMA types (26). The degree of agreement is determined as follows: poor when CCC <0.90, moderate between 0.90 and 0.95, substantial between 0.95 and 0.99, and almost perfect when >0.99 (27). Correlation analysis was used to verify the correlation between the two methods. Pitman's test was operated to exclude the proportional bias. Bland–Altman plots were constructed to visually display the consistency between BIA and DXA measurements. In addition, we investigated whether the difference between BIA and DXA was influenced by patients' height and SMA type which were shown in the scatter plots and verified by linear regression.

Microsoft Excel was used to collect data, and GraphPad Prism software was used to generate figures (version 6.01). The IBM SPSS software was used to conduct all statistical analyses (version 21.0). A *P*-value of <0.05 was considered statistically significant.

## Results

**Figure 1:**
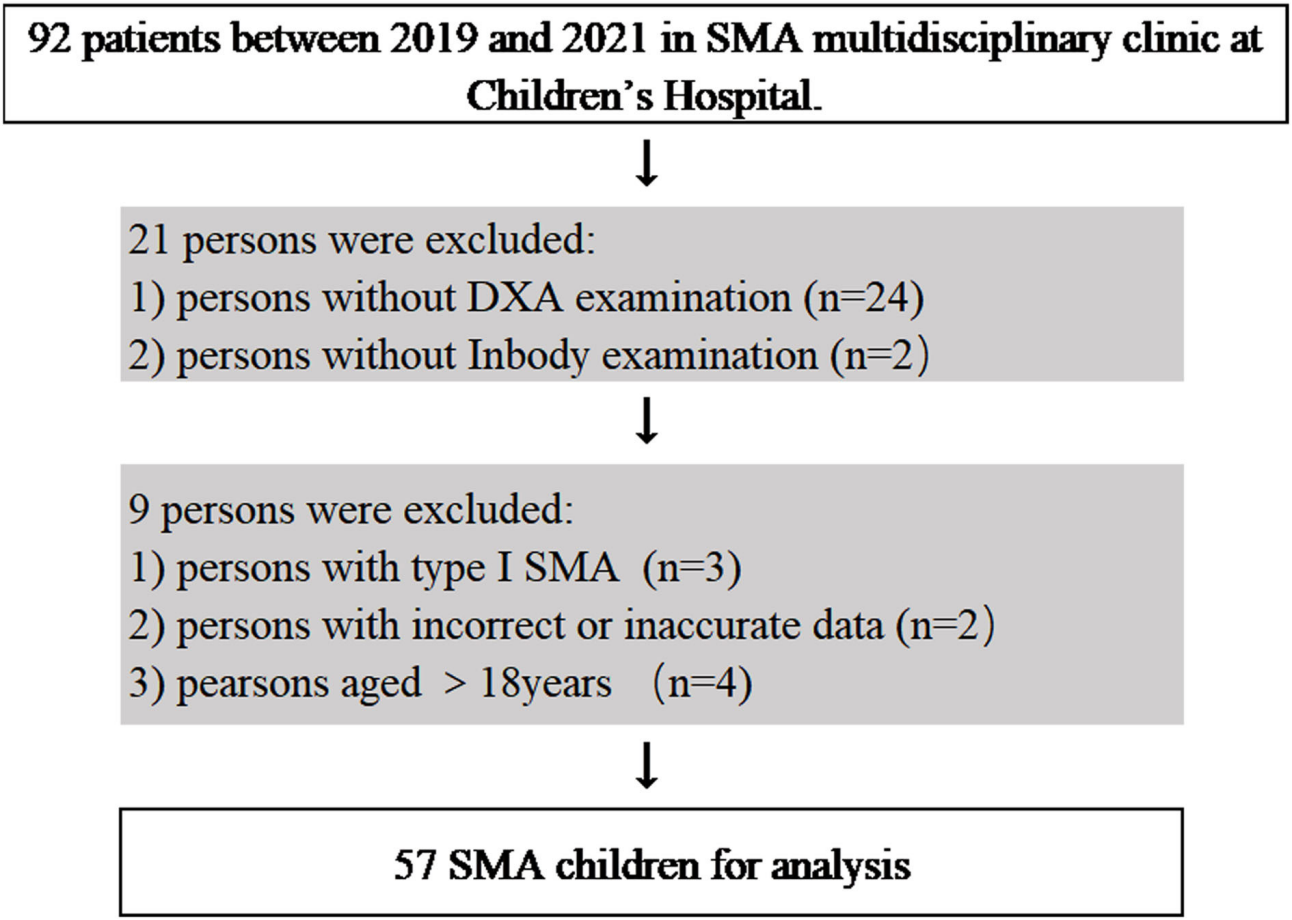
Flow chart of SMA children in MDT outpatients.

A total of 57 children with SMA (23 females and 34 males) were included in the analysis (Figure 1), with 27 of type II SMA and 30 of type III SMA. Table 1 shows the children's characteristics, puberty status, anthropometric measurements, ambulation status, HFMSE score, and degree of scoliosis. Children with type II and type III SMA showed average height and BMI z scores, respectively, which were lower than the mean values of the standard WHO growth charts. A specific growth curve was used to evaluate the BMI percentile in children with type II SMA. Twelve children's BMI percentiles were between 25% and 75%, whereas those of six children were below 25% and nine were above 75% (28). Children with the type II SMA included 1 nonsitter (3.7%) and 26 sitters (96.3%), and children with the type III included 7 sitters (23.3%) and 23 walkers (76.7%). The HFMSE scores of children with type II and III SMA were (8.3 ± 8.0) and (42 ± 15.8), respectively. The median (interquartile range) degree of scoliosis was 12 (18) degrees.

**Table 1:** Participants characteristics.

**Characteristic**	**SMA style**			***P-*value**
	**Overall (*n* = 57)**	**Type II (*n* = 27)**	**Type III (*n* = 30)**	
**Age, years**				
Mean ± SD	8.6 ± 3.4	7.5 ± 2.8	9.5 ± 3.6	0.09
Median (min, max)	7.4 (3.3,17.5)	6.8 (3.3,16.9)	8.4 (3.3,17.5)	
**Sex**, ***n*** **(%)**				
Male	34 (59.6%)	16 (59.3%)	18 (60.0%)	0.96
Female	23 (40.4%)	11 (40.7%)	12 (40.0%)	
**Puberty**				
Yes	11 (19.3%)	3 (11.1%)	8 (26.7%)	0.18
**Anthropometric measurements**				
Height (Mean ± SD, centimeter)	126.7 ± 17.1	120.7 ± 14.3	131.4 ± 17.8	0.03
Height for age Z score (HAZ)	−0.47 ± 1.28	−0.44 ± 1.43	−0.50 ± 1.17	0.76
Weight (Mean ± SD, kilogram)	27.8 ± 11.3	23.6 ± 7.4	31.2 ± 12.8	0.02
Body mass index (BMI)	16.7 ± 3.8	15.6 ± 3.2	17.5 ± 3.9	0.09
BMI for age Z score (BAZ)	−0.23 ± 2.3	−0.58 ± 2.28	0.05 ± 2.26	0.30
**Ambulation status**, ***n*** **(%)**				
Non-sitters	1 (1.8%)	1 (3.7%)	0 (0%)	<0.05
Sitters	33 (57.9%)	26 (96.3%)	7 (23.3%)	
Walkers	23 (40.4%)	0 (0%)	23 (76.7%)	
**Degree of scoliosis, degrees**				
Median (Interquartile Range)	12 (18)	13 (21)	10 (13)	0.10
**Motor function scale score**				
HFMSE score	26.0 (21.1)	8.3 (8.0)	42.0 (15.8)	<0.001

All body composition measurements by DXA and BIA, including MM, FM, BMC, and VFA, were presented in Table 2. On average, SMA type III patients had a higher MM than type II patients. Compared with DXA measurements, BIA measurements had a lower average FM (*P* <0.001) and higher average values of MM, BMC, and VFA (*P* < 0.001). On average, BIA underestimated the FM about 12.6% (Delta [BIA-DXA] = −1.6 kg) and overestimated MM by 11.9% (Delta [BIA-DXA] = 1.6 kg), BMC about 50% (Delta [BIA-DXA] = 0.5 kg), and VFA about 77.6% (Delta [BIA-DXA] = 43.5 cm). Overall, the difference between MM and FM measured by DXA and BIA was minor, whereas the difference between BMC and VFA was significantly large.

**Table 2:** Agreement between the measurement results of DXA and BIA.

	**Measurement (Mean** ±**SD)**		**Delta (BIA-DXA)**		**CCC (95% CI)**
	**DXA**	**BIA**	**Mean ±SD**	**95% LOA**	
**Muscle mass, kg**					
Overall (*n* = 57)	13.4 ± 5.6	15.0 ± 6.4	1.6 ± 1.8	−1.8 to 5.1	0.96 (0.93–0.98)
Type II (*n* = 27)	10.8 ± 2.8	11.5 ± 3.2	1.0 ± 1.7	−2.4 to 4.3	0.85 (0.70–0.93)
Type III (*n* = 30)	15.6 ± 6.5	17.7 ± 7.0	2.2 ± 1.6	−0.9 to 5.4	0.97 (0.94–0.99)
*P*-value	<0.01	<0.001			
**Fat mass, kg**					
Overall (*n* = 57)	12.7 ± 6.0	11.1 ± 5.9	−1.6 ± 1.8	−5.1 to 2.0	0.95 (0.92–0.97)
Type II (*n* = 27)	11.2 ± 4.1	11.8 ± 3.5	−1.0 ± 1.9	−4.7 to 2.8	0.90 (0.79–0.95)
Type III (*n* = 30)	14.1 ± 7.1	17.8 ± 7.2	−2.1 ± 1.5	−5.2 to 0.9	0.98 (0.95–0.99)
*P*-value	0.07	0.28			
**Bone mineral contents, kg**					
Overall (*n* = 57)	1.0 ± 0.4	1.5 ± 0.4	0.5 ± 0.4	−0.3 to 1.2	0.61 (0.42–0.75)
Type II (*n* = 27)	0.9 ± 0.5	1.6 ± 0.5	0.6 ± 0.4	−0.1 to 1.4	0.66 (0.38–0.83)
Type III (*n* = 30)	1.1 ± 0.4	1.5 ± 0.4	0.3 ± 0.3	−0.3 to 1.0	0.66 (0.40–0.83)
*P*-value	0.08	0.41			
**Visceral fat area, cm**					
Overall (*n* = 57)	56.0 ± 24.3	99.5 ± 50.2	43.5 ± 37.8	−30.7 to 117.6	0.54 (0.33–0.70)
Type II (*n* = 27)	57.9 ± 24.0	105.2 ± 49.5	47.3 ± 41.2	−33.4 to 128	0.44 (0.08–0.70)
Type III (*n* = 30)	54.4 ± 24.9	94.4 ± 51.2	40.0 ± 34.9	−28.3 to 108.4	0.62 (0.35–0.80)
*P*-value	0.59	0.42			

Agreement was assessed overall and according to the SMA type. The mean difference between the two methods (BIA-DXA) was compared to zero using the one-sample t-test, and 95% limits of agreement were calculated as the mean difference ± 1.96 SD. In addition, Lin's concordance correlation coefficient was computed with a 95% confidence interval calculated using Fisher's z transformation.

DXA, Dual-energy X-ray Absorptiometry; BIA, Bioelectrical Impedance Analysis; LOA, limits of agreement, CCC, Concordance correlation Coefficient; C.I, Confidence Intervals.

**Figure 2:**
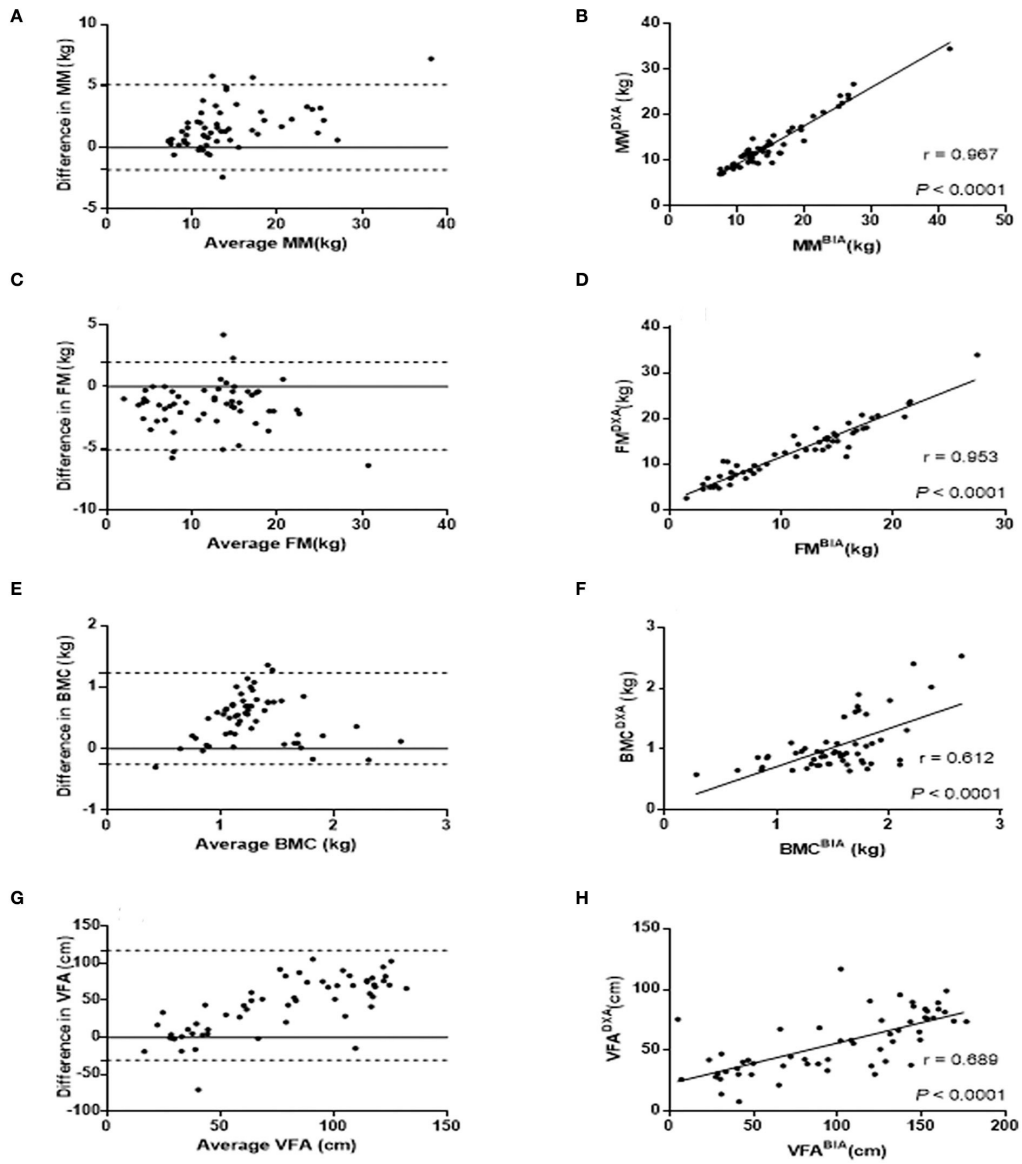
Comparison of MM, FM, BMC, and VFA measurements by DXA and BIA. Bland-Altman plots were constructed with the difference of MM **(A)**, FM **(C)**, BMC **(E)**, and VFA **(G)** between DXA and BIA. The differences between the two methods are plotted based on their mean values. The dashed line represents the 1.96 SD value of the two methods. Correlation analysis were applied to identify the correlation of MM **(B)**, FM **(D)**, BMC **(F)**, and VFA **(H)** between DXA and BIA with correlation coefficient r shown in the scatter plots. FM, Fat mass; MM, Muscle mass; BMC, Bone mineral content; VFA, Visceral fat area.

The CCC for body composition measurements by DXA and BIA ranged from 0.54 to 0.96. There are substantial correlations of MM CCC = 0.96 (95% CI = 0.93–0.98) and FM CCC = 0.95 (95% CI = 0.92–0.97) measurements, and poor correlation of BMC CCC = 0.61 (95% CI = 0.42–0.75) and VFA CCC = 0.54 (95% CI = 0.33–0.70) measurements between DXA and BIA in children with SMA. Those correlations in whole participants were consistent with those in children with type III SMA. Moreover, patients with type II SMA had a lower CCC of body composition measurements than patients with type III. There were moderate correlations of MM and FM measurements, and poor correlation of BMC and VFA measurements between DXA and BIA in children with type II SMA. Pitman's tests showed that there is no statistical significance of proportional bias for MM, FM, and BMC (*P* = 0.056, 0.622, and 0.850, respectively), but the VFA-related results were statistically significant (*P* < 0.05). The Bland–Altman analysis suggests the majority of participants were within LOA. Furthermore, the correlation analysis verified the positive correlation of MM (r = 0.967, *P* < 0.0001), FM (r = 0.953, *P* < 0.0001), BMC (r = 0.689, *P* < 0.0001), and VFA (r = 0.689, *P* < 0.0001) between the two methods (Figures 2B,D,F,H). The Bland–Altman analysis and scatter plots based on the patients with type II and type III SMA were shown in Supplementary Figures S1, S2, respectively. Although the average difference between BIA and DXA measurements on the same patient was either significantly lower or significantly higher than zero, the variance of this difference was significantly higher than the average. As a result, while BIA measurements had some bias, the 95% LOA for the difference between BIA and DXA measurements on an individual patient was usually zero (Figures 2A,C,E,G).

**Figure 3:**
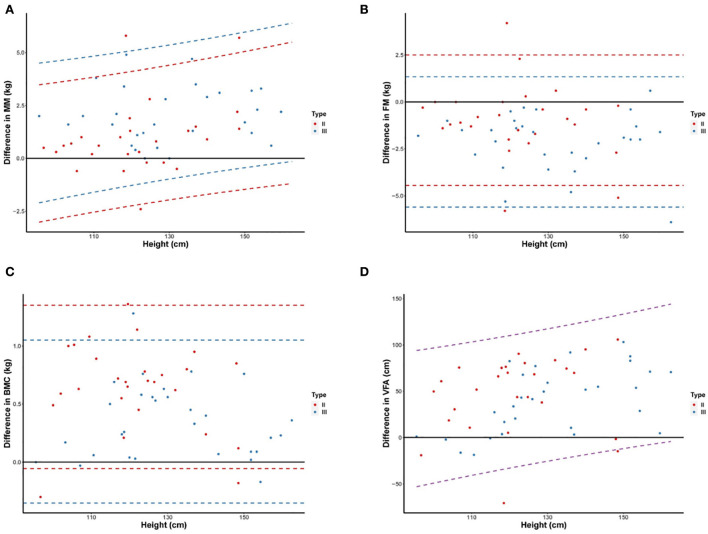
Differences of MM **(A)**, FM **(B)**, BMC **(C)**, and VFA **(D)** measurements between DXA and BIA according to increasing height. Scatter plots and 95% LOA between the difference of values by DXA and BIA and height were shown for children with type II and III SMA. The difference in values is BIA minus DXA. The dashed line represents the 95% LOA of values, different colors were used to indicate children with type II (red), type III (blue), and all SMA (purple). FM, Fat mass; MM, Muscle mass; BMC, Bone mineral content; VFA, Visceral fat area; LOA, limits of agreement.


Linear regression analysis showed that measurement differences for MM and VFA increased with an increase in patients' height, whereas differences for FM and BMC did not differ with height (Figure 3). In addition, measurement differences for MM, FM, and BMC differed with SMA type, whereas differences for VFA were similar between SMA types. Linear regression is based on formulas for predicting measurement differences for MM, FM, BMC, and VFA as a function of height and/or SMA type as follows:

$$\begin{array}{l} \text{Difference in MM}=-2.5+0.03\times \text{height }\left(\text{cm}\right)+0.97\\ \;\times \text{SMAtypeIII}\\ \text{Difference in FM}=-0.97-1.16\times \text{SMA type III}\\ \text{Difference in BMC}=0.65-0.30\times \text{SMA type III}\\ \text{Difference in VFA}=-50.0+0.74\times \text{height }\left(\text{cm}\right). \end{array}$$

## Discussion

This present study compared the concordance between the BIA device and the current gold-standard DXA device on the measurement of body composition in children with SMA for the first time. We put forward that, although BIA overestimates the MM, BMC, and VFA, and underestimates the FM in SMA, compared with DXA, both of them have good consistency for measuring MM and FM, especially in patients with type III. Hence, BIA could be considered as a portable, simple-to-use, and appropriate method for body composition measurement to guide clinical nutritional assessment in patients with SMA, particularly when measuring MM and FM. Studies have compared body composition measurements assessed using BIA and DXA in various populations, including healthy individuals (23), athletes (29), overweight and obese children (30), and adults (24). These studies showed that MF-BIA can underestimate FM and overestimate fat-free mass in an obese population compared with DXA (31–33). However, because SMA is a rare neuromuscular disease, few studies have focused on children with SMA. A study involving a small sample of children with SMA found that BIA determined using Cordain's equation has high sensitivity and specificity for screening overweight people (34). We also discovered findings of studies on other primary neuromuscular diseases (35). Consistent with our research, L. Ellegård's study showed that children with primary neuromuscular disorders had proportionally more FM and less MM than the general population, regardless of normal or abnormal BMI, and that MF-BIA overestimated MM with a systematic bias (35). Another French study found no significant differences between BIA and DXA estimates, and they could be used to follow-up the dynamic changes in the nutritional status of ambulatory pediatric patients with Duchenne muscular dystrophy (36). Similar findings were reported using labeled water dilution (WD) as the reference method. FM estimates by BIA were close to those by WD and were used in the early detection of fat accumulation to prevent obesity (37).

Several reasons may explain the discrepancies between body composition estimates reported in these studies. First, hydration could have been a potentially important factor. The hydration of higher lean soft tissue or a larger proportion of extracellular fluid (ECF) may lead to a greater overestimation of MM by BIA than by DXA (38). Second, acute fluid or food ingestion also elevates body mass and impedance, contributing to higher FM calculations (39). Third, anthropometric variables, such as circumferences and lengths, may have effects on the DXA and BIA estimates in different aspects because the influences of X-ray imaging technology and the flow of electrical currents brought by such variables may differ from each other (38). For children with SMA who use a wheelchair or have scoliosis, the height was replaced with arm span, which has a greater measurement error. Furthermore, BIA differences vary with BMI because of a lack of accuracy in the extremely low BMI class (23). Some children with SMA, particularly malnourished ones, have low BMI.

There is no denying that DXA, as the gold-standard method for evaluating body composition, takes advantage of BMC and density measurements to obtain knowledge of bone health (40). Nevertheless, DXA has several limitations for children with SMA: 1) The DXA method requires patients to be positioned in a fixed scan zone for 10 min, which necessitates good cooperation from children. Some children are unable to complete the coordination without the assistance of an anesthetist, 2) Children with SMA and severe scoliosis may cross the scanning table's borders, affecting the accuracy of the results. Due to mild radiation, DXA examinations should be limited to no more than two per year. In contrast, dietitians are more interested in tracking dynamic alterations in body composition rather than absolute values. Repeated nutrition assessments are required for children with SMA, particularly those with malnutrition or obesity or those who are taking drug therapy. Additionally, MF-BIA machines may be less susceptible to the deviation caused by redistribution between extracellular and intracellular water, which is probably a relatively superior approach for assessing body composition (41, 42). The BIA phase angle (PhA) reflects the ratio of intracellular to extracellular water and is significantly lower in patients with neuromuscular disease (43). Phase angle (PhA) may be used as a surrogate measure of MM because it correlates well-with the clinical staging of primary neuromuscular disease (35). BIA has limitations as well. BIA overestimates the MM among different populations compared with DXA. There are several potential effects resulting from the electrical model's shape, the properties of the cell membrane, and the fraction of the current entering the intracellular space at various frequencies, such as non-standardization of body position, previous physical exercise, and food or fluid intake (37). These constraints may not preclude longitudinal comparisons, as the deviations may cancel each other out. Additionally, VFA measurement has a huge difference between BIA and DXA, since VFA cannot be measured directly and the regression formula for the health population is not appropriate for patients with SMA. So far, there is no accurate validation of segmental BIA in conditions of rare diseases, such as children with SMA.

### Strengths and limitations

To the best of our knowledge, this is a study with the largest sample size that compared body composition measurements obtained using DXA and BIA in children with SMA, although previous studies in healthy children or children with other neuromuscular diseases have been conducted (33, 44). Furthermore, because food and fluid intake influence body composition estimates (20), this study ensured a research prerequisite of food and fluid abstention lasting for an overnight period (45). One limitation of our study is that we did not include children with type I because the majority of them do not survive beyond the first 2 years of life without intervention. We anticipated recruiting more children with type I because Nusinersen improved survival in children with type I SMA (46). The second limitation is that we only used one MF-BIA device with a proprietary equation based on healthy children, and the device was most likely not adapted to children with SMA. We hope to develop body composition formulas for some rare diseases in future.

With the widespread use of drugs and gene therapy, an increasing number of children with SMA will have a higher survival rate and quality of life. The ability to accurately estimate MM and FM is of vital importance for assessing the nutritional status of children with SMA. BIA is emerging as an available approach in routine clinical practice for evaluating and monitoring nutrition.

## Conclusion

To sum up, BIA overestimates MM and underestimates FM, BMC, and VFA in children with type II and type III SMA, compared with the gold standard DXA measurement. Apart from this, FM and MM measured by BIA and DXA in children with type II and III SMA are in good agreement, whereas BMC and VFA are not, and this consistency is more obvious in children with type III than that in children with type II. In a word, BIA is considered to be a non-invasive, easy-to-use, and repeatable measuring tool for monitoring FM and MM, which is expected to be widely used in children with SMA in clinical practice.

## Data availability statement

The raw data supporting the conclusions of this article will be made available by the authors, without undue reservation.

## Ethics statement

The studies involving human participants were reviewed and approved by the Ethics Committees of the Children's Hospital and Zhejiang University School of Medicine (No. 2019-IRB-171). Written informed consent to participate in this study was provided by the participants' legal guardian/next of kin.

## Author contributions

WW contributed to the data analysis and writing of the manuscript. YF, QL, FC, and YC contributed to the collection of the data. SM and MM contributed to the design of the experiment and revising of the manuscript. All authors critically revised the manuscript, and read and approved the final manuscript.

## Funding

This study was supported by the National Natural Science Foundation of China (Grant No. 82271735), Key R&D Program of Zhejiang Province (2022C03167), the Zhejiang Province Public Welfare Technology Application Research Project (LGC21H090001), the Key Technologies Research and Development Program of Zhejiang Province (2021C03099), and a project supported by Scientific Research Fund of Zhejiang University (XY2022045).

## Conflict of interest

The authors declare that the research was conducted in the absence of any commercial or financial relationships that could be construed as a potential conflict of interest.

## Publisher's note

All claims expressed in this article are solely those of the authors and do not necessarily represent those of their affiliated organizations, or those of the publisher, the editors and the reviewers. Any product that may be evaluated in this article, or claim that may be made by its manufacturer, is not guaranteed or endorsed by the publisher.

## Abbreviations

SMA: Spinal muscular atrophy
DXA: X-ray absorptiometry
BIA: Bioelectrical impedance analysis
FM: Fat mass
MM: Muscle mass
BMC: Bone mineral content
VFA: Visceral fat area
CCC: Concordance correlation coefficient
MDT: Multidisciplinary team
BMI: Body mass index
FM: Fat mass
CT: Computed tomography.
