# Supplementary material for: A comparative analysis of body composition assessment by BIA and DXA in children with type II and III spinal muscular atrophy
Source: Front Neurol. 2022 Nov 18;13:1034894. doi: 10.3389/fneur.2022.1034894 (PMC9715747; doi:10.3389/fneur.2022.1034894)
Supplement: Supplementary file 1 [file Data_Sheet_1.PDF]

Supplementary Figures

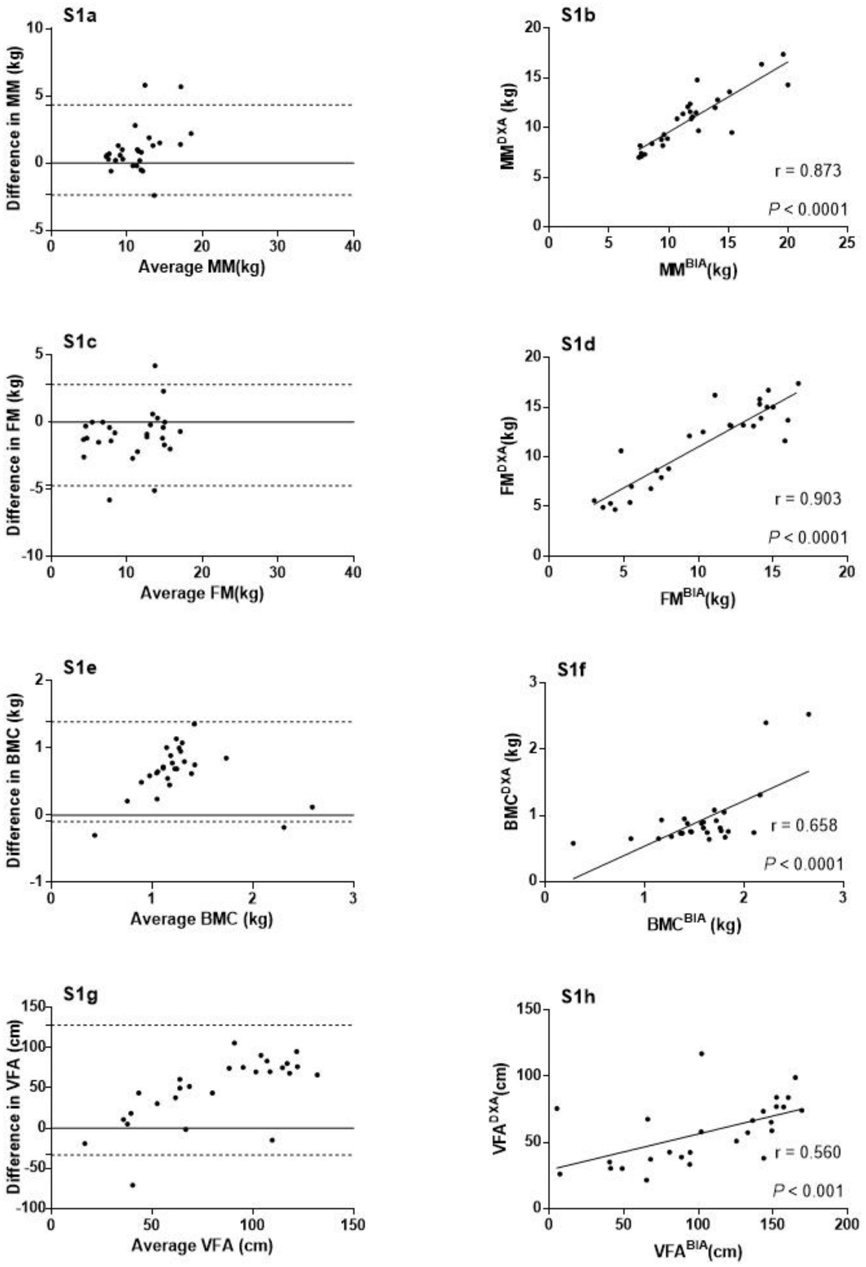

Supplementary Figure 1. Comparison of MM, FM, BMC, and VFA measurements by DXA and BIA for type II SMA children.*

*Bland–Altman plots were constructed with difference of MM, FM, BMC, and VFA between DXA and BIA. The differences between the two methods are plotted based on their mean values. The dashed line represents the 1.96 SD value of the two methods. Scatterplots and correlation coefficient r between values of DXA and BIA also were shown.

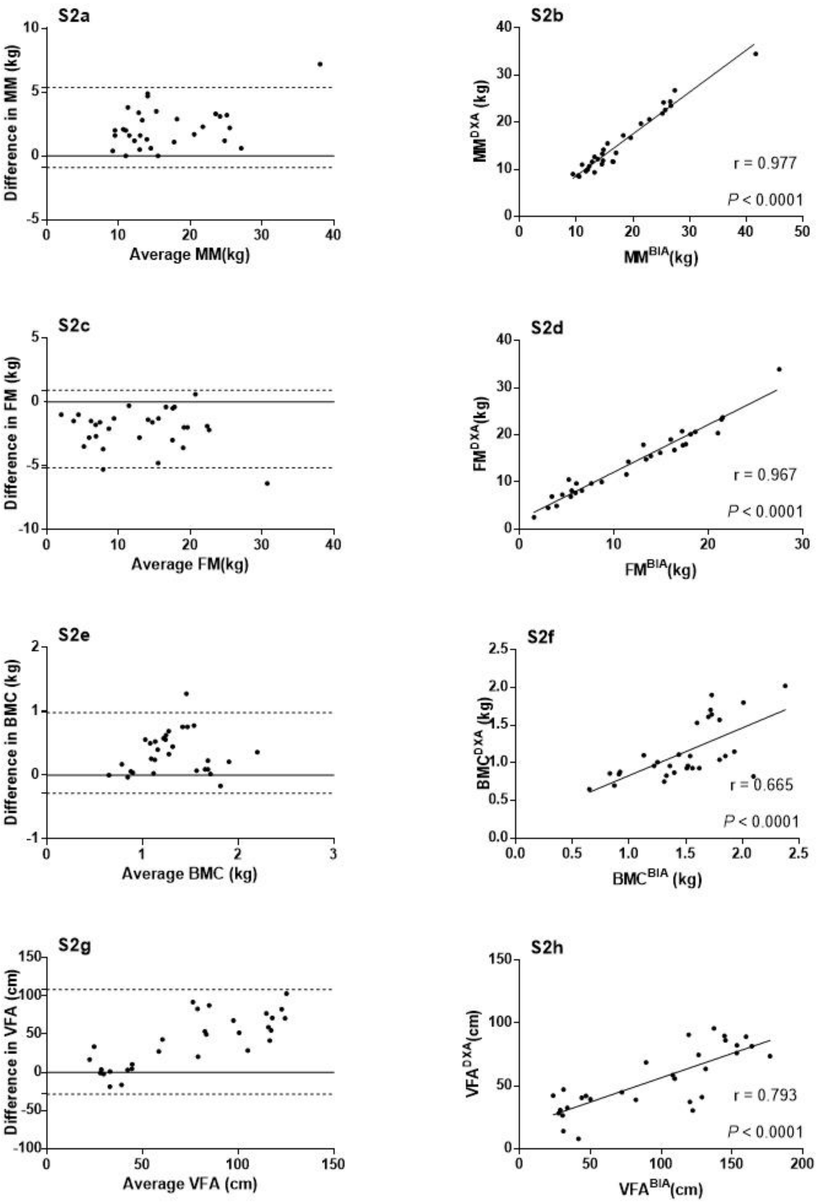

Supplementary Figure 2. Comparison of MM, FM, BMC, and VFA measurements by DXA and BIA for type III SMA children.*

*Bland–Altman plots were constructed with difference of MM, FM, BMC, and VFA between DXA and BIA. The differences between the two methods are plotted based on their mean values. The dashed line represents the 1.96 SD value of the two methods. Scatterplots and correlation coefficient r between values of DXA and BIA also were shown.
